# A Nonlinear Dynamical View of Kleiber’s Law on the Metabolism of Plants and Animals

**DOI:** 10.3390/e26010032

**Published:** 2023-12-28

**Authors:** Luis Jovanny Camacho-Vidales, Alberto Robledo

**Affiliations:** Instituto de Física, Universidad Nacional Autónoma de México, Apartado Postal 20-364, Mexico City 01000, Mexico

**Keywords:** Kleiber’s law, allometry, nonlinear dynamics, complex systems, statistical mechanics

## Abstract

Kleiber’s empirical law, which describes that metabolism increases as the mass to the power 3/4 , has arguably remained life sciences’ enigma since its formal uncovering in 1930. Why is this behavior sustained over many orders of magnitude? There have been quantitative rationalizations put forward for both plants and animals based on realistic mechanisms. However, universality in scaling laws of this kind, like in critical phenomena, has not yet received substantiation. Here, we provide an account, with quantitative reproduction of the available data, of the metabolism for these two biology kingdoms by means of broad arguments based on statistical mechanics and nonlinear dynamics. We consider iterated renormalization group (RG) fixed-point maps that are associated with an extensive generalized (Tsallis) entropy. We find two unique universality classes that satisfy the 3/4 power law. One corresponds to preferential attachment processes—rich gets richer—and the other to critical processes that suppress the effort for motion. We discuss and generalize our findings to other empirical laws that exhibit similar situations, using data based on general but different concepts that form a conjugate pair that gives rise to the same power-law exponents.

## 1. Introduction

At least two of the kingdoms of biology on earth, plant and animal, seem to have found sustainable coexistence over an extended period of time. This is perhaps best quantified via the metabolic rates of organisms. When these rates are sorted out according to their mass, a robust scaling relation emerges, a power law with an exponent close to 3/4 spanning several orders of magnitude for both kingdoms. This is known as Kleiber’s law [1,2,3,4] or, more generally, allometric scaling [5,6,7,8,9,10,11,12,13,14,15]. Since its discovery, this scaling law has attracted attention, and many attempts have been put forward towards its understanding [5,6,7,8,9,10,11,12,13,14,15]. One instance is to consider dissipation via a surface-to-volume ratio that indicates a slightly different value for the exponent, 2/3 [5,15,16]. Other more structured developments are (i) a branching scheme for plants with unassisted conveyance of raw materials and nutrients [6,7,14] and (ii) a set of scaling laws for animals that require a pump to propel raw materials and nutrients [6,9,10,11]. In our case, we look for a general principled conjugate pair of kingdom universality classes without reference to mechanisms but linked to a nonlinear dynamical approach that, in turn, can be couched in the language of statistical mechanics.

Over the last few years, we have developed a general theoretical procedure [17,18,19] to quantitatively reproduce the distributions of many real types of ranked data. The approach is based on dissipative nonlinear dynamics of low dimensionality. See also in [17,18,19] earlier references on how our approach developed. We specifically consider iterated maps at or near a tangent bifurcation [20,21]. A central role is played by the renormalization group (RG) fixed-point map $f^{*}\left(x\right)$ for the route out of chaos known as intermittency [21,22,23]. A brief recall [21,22,23] for the derivation of $f^{*}\left(x\right)$ is to consider that a generic (one-dimensional) map in the neighborhood of tangency at x=0 with the identity function reads,

(1) $$f\left(x\right)=x+{u|x|}^{z},$$

where we omitted higher-order terms, *u* is a constant, and the power *z* defines the nonlinearity at tangency. The customarily applied [21,22] RG transformation for this nonlinear dynamical route to (or out of) chaos is the functional composition f(f(x)) ; the RG flow occurs in the space of functions tangent to the identity; and its RG fixed-point, the map $f^{*}\left(x\right)$ , satisfies

(2) $$f^{*}\left(f^{*}\left(x\right)\right)=\gamma^{-1}f^{*}\left(\gamma x\right),$$

where the scaling parameter γ is to be determined, while the first two terms of the expansion of $f^{*}\left(x\right)$ must reproduce f(x) in Equation (1). The fixed-point map $f^{*}\left(x\right)$ was obtained in analytical closed form by Hu and Rudnick over 40 years ago [22]. This is

(3) $$f^{*}\left(x\right)=x\exp_{z}\left(ux^{z-1}\right),$$

where $x^{z-1}\equiv \mathrm{sign}\left(x\right){|x|}^{z-1}$ , and where $\exp_{z}$ is the *q*-deformed exponential function, $\exp_{q}\left(x\right)\equiv {\left[1+\left(1-q\right)x\right]}^{1/(1-q)}$ . The scaling parameter is $\gamma =2^{1/(z-1)}$ . All the trajectories $x_{t}$ , t=0,1,2,..., of $f^{*}\left(x\right)$ have the form [23]

(4) $$x_{t}=x_{0}\exp_{z}\left(x_{0}^{z-1}ut\right).$$

That is, for all *z*, *u*, and $x_{0}$ , any pair of trajectories can be transformed into each other via appropriate rescaling of these parameters. Interestingly, as we describe here, the tangency feature of $f^{*}\left(x\right)$ present for z≥2 transforms below z=2 , first, into a cusp and then into a different map shape relevant to our description of Kleiber’s law below.

It is worth mentioning that the fixed-point maps $f^{*}\left(x\right)$ for the other (and only) two routes to (or out of) chaos, period doubling and quasi-periodicity [24], were originally obtained numerically via approximations of their power series representation [21]. Their analytical closed-form expressions, also in terms of the *q*-exponential function $\exp_{q}\left(x\right)$ , have become known only very recently [25]. The inverse function of the *q*-exponential, the *q*-logarithm, is given by $\ln_{q}\left(x\right)\equiv \left[x^{1-q}-1/\left(1-q\right)\right]$ . Both functions reduce, respectively, to the ordinary exponential and logarithmic functions when q=1 . The latter pair of functions plays a central role in ordinary statistical mechanics, while the *q*-deformed pair is correspondingly central for the Tsallis generalized statistical mechanics [26,27]. When the deformation parameter *q* (the nonlinearity *z* in $f^{*}\left(x\right)$ ) falls within 1<q<∞ , both $\exp_{q}\left(x\right)$ and $\ln_{q}\left(x\right)$ asymptotically approach power laws.

Actually, the origin of the rank distributions approach was expressed in a stochastic process language [28], but we provided a precise analogy [18] that converts the random variable description of the ranked data sample into a deterministic iterated map trajectory, $x_{t}$ , t=0,1,2... , for the same data. The starting point in the stochastic approach is a parent (or source) probability distribution P(N) for the data samples of magnitudes *N*. The parent distribution is assumed to take the form of a power law $P\left(N\right)=aN^{-\alpha }$ , *a* being a constant factor, α>1 , together with the limits α=1 and α→∞ , hyperbolic and exponential decay, respectively. The rank distributions are obtained from the parent distribution P(N) via integration. First, obtain the complementary cumulative distribution $\Pi (N\left(k\right),N_{\max})$ of P(N) ,

(5) $$\Pi \left(N\left(k\right),N_{\max}\right)=a\int_{N(k)}^{N_{\max}}N^{-\alpha }dN,$$

where the magnitudes in a sample with N items are sorted out starting with the largest, $N_{\max}$ , and continuing with decreasing magnitudes down to N(k) , and where k=0,1,2,... , is the rank variable, with k=0 for $N_{\max}$ . We call the function N(k) the size-rank distribution, though technically, it is a quantile [29]. On the other hand, the rank *k* is equal to NΠ so that Equation (5) becomes

(6) $$\ln_{\alpha }N\left(k\right)=\ln_{\alpha }N_{\max}-{\left(aN\right)}^{-1}k,$$

where we used the *q*-deformed logarithm expression. The size-rank distribution N(k) is explicitly obtained from Equation (6) by making use of the *q*-deformed inverse functions. This is

(7) $$N\left(k\right)=N_{\max}\exp_{\alpha }\left[-N_{\max}^{\alpha -1}\;{\left(aN\right)}^{-1}k\right].$$

The translation from the language of rank distributions into that for the trajectories of the RG fixed-point map $f^{*}\left(x\right)$ is obtained via t=k , $x_{0}=-N_{\max}$ , $x_{t}=-N\left(k\right)$ , u=1/aN , and z=α [17,18,19]. Notice that the trajectory $x_{t}$ that translates into N(k) takes place at the left x<0 of the point of tangency x=0 . Furthermore, the map that corresponds to the parent distribution P(N) , the starting point, is given by Equation (1), rewritten as [18]

(8) f(x)=x+u/P(−x).

In the following Section 2, we succinctly present our approach to reproduce rank distributions of very diverse kinds with emphasis on the features that are prominent to our consideration of Kleiber’s law. These are universality classes indicated by the values of the exponent α (also denoted as the deformation *q* or the nonlinearity *z*, α=q=z ). In particular, we focus on the location of the conjugate pairs (q,Q) , values where the deformed exponential and its inverse function, the deformed logarithm, share the same power law decay. When referring to these pairs, we write *q* for $\exp_{q}$ and *Q* for $\ln_{Q}$ . These pairs include a limit for validity of ordinary statistical mechanics (q=1,Q→∞) , the frequency and magnitude coincidence for Zipf’s law [30] (q=2,Q=2) , and other cases mentioned below. In the next Section 3, we extend the approach to incorporate rates of change of key quantities, as it is the case of metabolism in biology. As we shall see, this extension involves the consideration of the RG fixed-point map for the tangent bifurcation into a different regime (that for values of the nonlinearity z<2 ). In Section 4, we present our results for Kleiber’s law as derived from our formalism by specific choices of universality classes that represent the guiding principle of each biological kingdom. Finally, in Section 5, we discuss our results in connection with the Tsallis generalized entropy.

## 2. Rank Distributions and Their Universality Classes

Importantly, particularly for our purposes here, there is a well-defined conceptual distinction concerning rank distributions, on the one hand, those referring to magnitudes, sizes, and, on the other hand, those referring to frequencies, occurrences. According to our approach [17], the former, N(k) , k=0,1,2,⋯ , is given by Equation (7), while the latter, denoted as F(k’) , k’=0,1,2,⋯ , is given by

(9) $$F\left(k’\right)=aN\left[\ln_{\alpha }N_{\max}-\ln_{\alpha }k’\right],$$

where we have rewritten Equation (6) by introducing the changes of the variables F=NΠ and k’=N . The non-normalized frequency-rank distribution F(k’) is often used as it is constructed directly from the numbers of occurrences in data samples. These functions are inverses of each other and asymptotically exhibit the same power-law exponent ζ=−1 with q=Q=2 for the Zipf class (city sizes or moon crater diameters obey the same power law as occurrences of words or earthquake frequencies) [19]. Interestingly, when α=q=Q=2 , the asymptotic power-law rank interval for both the *q*-exponential and the *Q*-logarithm displays the same exponent ζ=1/(1−q)=(1−Q)=−1 .

Typically, ranked finite data samples show power-law decay only through an intermediate rank interval with different conducts for small and large ranks. The prevailing focus of interest in this central power-law interval in real finite data rank distributions and not on the small and large rank deviations from the power law led to the same identification as Zipf’s law for both magnitude and frequency ranked data samples. However, we can clearly distinguish between these two qualities in our formalism [17]. Additionally, we can choose a parent distribution from the start to represent ‘frequency’ instead of ‘magnitude’ and find that the values of *q* and *Q* appear interchanged [17]. Alternatively, we can use the precise analogy that exists between the trajectories of the RG fixed-point map $f^{*}\left(x\right)$ with the rank distributions derived from a parent distribution P(N) . As we have seen, our approach leads to rank distributions expressions in terms of *q*-exponential and *q*-logarithmic functions. These expressions reproduce real behavior for small rank, whereas the finite-size effect observed for large rank is also obtained quantitatively simply via the shift of the map $f^{*}\left(x\right)$ off tangency [18]. Ordinary exponential decay of rank distributions occurs for the pair of exponents α=1 and α→∞ for both magnitudes and frequencies. All other values of (1<α<∞) lead to rank distributions of the form in Equations (7) and (9) [17,18,19].

We now assign some meaning, backed by real data examples, to natural numbered values of the exponent α=z=q . In the limit when α=1 , hyperbolic P(N) and linear iterated map, we obtain exponential decay for N(k) consistent with the ordinary statistical mechanics q=1 . See the comment at the end of this Section. Magnitudes and frequencies take values within real number intervals without restriction (a real data case we have analyzed is that of infant mortality [18]). We have also shown that this case applies to Benford’s first digit law [17]. When α=2 , we have a borderline case that corresponds to the classical Zipf’s law [30]. There are many real data examples that illustrate this circumstance [18,19]. Magnitudes (or frequencies) do not fill real number intervals but much less, like an infinite numerable set (e.g., an infinite vocabulary). When α=2 , the map f(x) is tangent to the identity line with nonzero curvature and trajectories develop hyperbolic power-law behavior, ζ=−1 , near tangency. For an ample discussion of this borderline case, see Ref. [19]. For α=3 , we have selective behavior that corresponds to rich-gets-richer processes that is analogous to preferential attachment network growth [31,32]. This is represented by a map f(x) with cubic tangency with the identity line. We end this list with α=4 , when f(x) displays vanishing curvature at tangency with the identity line, a circumstance analogous to critical point behavior where displacements in the neighborhood of tangency (or criticality) have (thermodynamic potential) vanishing cost [33].

**Figure 1:**
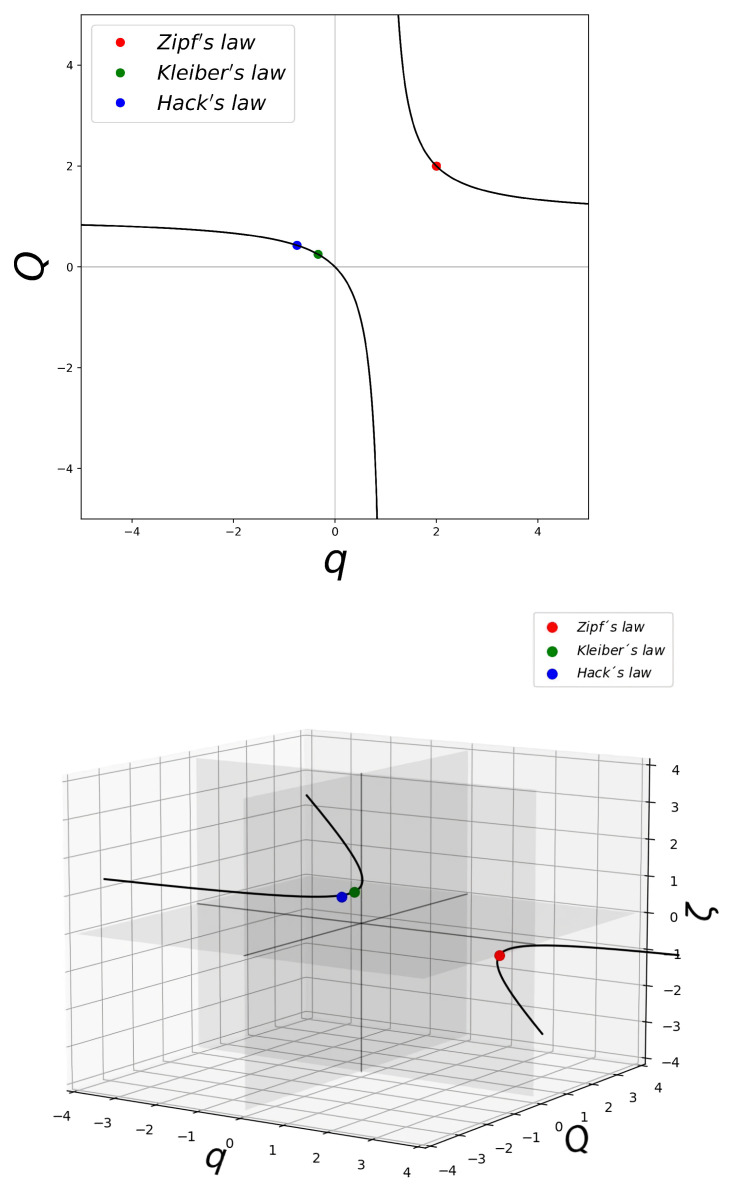
Upper panel shows the locus for identical power-law exponent ζ shown (within an interval of the independent variable) by the *q*-exponential function and its inverse function, the *Q*-logarithm. That is, ζ=1/(1−q)=1−Q . There are two mirror branches. The dots show the values of the conjugate pairs (q,Q) relevant for Zipf’s law (2,2) , Kleiber’s law (−1/3,1/4) , and Hack’s law (−1/2,1/3) . See text. Lower panel. The same as above but a three-dimensional rendering that shows the value of the power-law exponent ζ . See text.

We have made a clear distinction between data that result from quantities related to the consideration of sizes or magnitudes and data produced by temporal behaviors that manifest as frequencies. We turn our attention now to the occurrence of conjugate universality classes (given by pairs of specific values of α ) that asymptotically generate power-law scaling laws that have the same exponents. Here, we point out examples for rank distributions, but in the next section, we focus on dissipation or other rates such as in the case of Kleiber’s law. These are shown in Figure 1, where the *q*-exponential (magnitudes) exhibits the same power-law exponent ζ as its inverse function, the *Q*-logarithm (frequencies). The (q,Q) -indexes for this condition satisfy the simple relation ζ=1−Q=1/(1−q) . A prominent case we have already pointed out is Q=q=2 , the borderline case [19] for the empirical Zipf’s law. Another example corresponds to the Boltzmann–Gibbs statistics. When q=1 , the *q*-exponential and *q*-logarithmic functions become the ordinary exponential and logarithmic functions, respectively. Likewise, when q=1 , Tsallis entropy reduces to the Boltzmann–Gibbs or Shannon expression. See Refs. [26,27] for an extended description. The value we have quoted for α=q=1 , the Fibonacci number set [19] (illustrated by infant mortality [18]), is conjugate to Q→∞ displayed by the factorial number set [19] (illustrated by gun ownerships per capita [18]). Additionally, when Q→∞ the *q*-exponential and the *q*-logarithm become the ordinary exponential and logarithmic functions, but with the roles interchanged.

## 3. Scaling of Rates and Characteristic Times and Their Universality Classes

We extend here our formalism for rank distributions to incorporate in it the determination of other important quantities. Specifically, we consider now the concept of rate, or equivalently, its reciprocal, the characteristic time, relevant, for instance, to Kleiber’s law. The particular example of interest here is the metabolic *M* (or energy dissipation) rate of organisms as a function of the individual mass or volume *N*. We start with the parent or source probability distribution for the metabolism of a living organism P(M) , where *M* shall be considered to be a function of the organism size *N*. If metabolic rate values are to span real number intervals compatible with ordinary statistical mechanics, we have $P\left(M\right)\;=aM^{-1}$ , *a* being a constant factor. Recall that α=1 returns the ordinary exponential and logarithmic functions to the rank distribution expressions Equations (7) and (9). This is similar to the exponential decay of configurational distributions, and access via the logarithm to thermodynamic potentials form partition functions in ordinary statistical mechanics.

We now particularize the parent distribution to a specific universality class α≥1 , e.g., a kingdom in biology. We consider then the parent distribution $P\left(R\right)=cR^{\alpha }$ , *c* being a constant, for an energy dissipation rate, or metabolic rate, *R*. Here, the size or magnitude is the reciprocal, the characteristic time $T=R^{-1}$ . As we pointed out, the value of α , which specifies the universality class, carries a general meaning. This choice determines not only the form of the rank distributions but also, as we see now, rates such as *R*. As a consequence of the two parent distributions, P(M) and P(R) , we have introduced a new function, R(M) , that follows the power law $R\left(M\right)=bM^{-1/\alpha }$ . We have the following differential equation:

(10) $$\frac{dM}{dN}=R\left(M\left(N\right)\right)=bM^{-\frac{1}{\alpha }}\;,$$

with b=a/c . Considering that the use of data for metabolism to illustrate Kleiber’s law is sorted out from small to large organism mass or volume *N*, we integrate the above to obtain the cumulative metabolic rate μ(M(N)) :

(11) $$\mu \left(M\left(N\right)\right)=\int_{M_{0}}^{M(N)}R\left(M^{\prime }\right)dM^{\prime }=b\int_{M_{0}}^{M(N)}M^{\prime -1/\alpha }dM^{\prime }\;.$$

If μ(M(N)) is normalized, this is equal to N/bN , where N is the sum total of sizes in the data sample. After integration, we have

(12) $$\frac{N}{bN}=\frac{1}{1-\alpha^{-1}}M{\left(N\right)}^{1-\alpha^{-1}}-\frac{1}{1-\alpha^{-1}}M_{0}^{1-\alpha^{-1}}=\ln_{\alpha^{-1}}M\left(N\right)-\ln_{\alpha^{-1}}M_{0}$$

and solving for M(N) , we have

(13) $$M\left(N\right)=M_{0}\exp_{\alpha^{-1}}\left[M_{0}^{\alpha^{-1}-1}\;{\left(bN\right)}^{-1}N\right].$$

**Figure 2:**
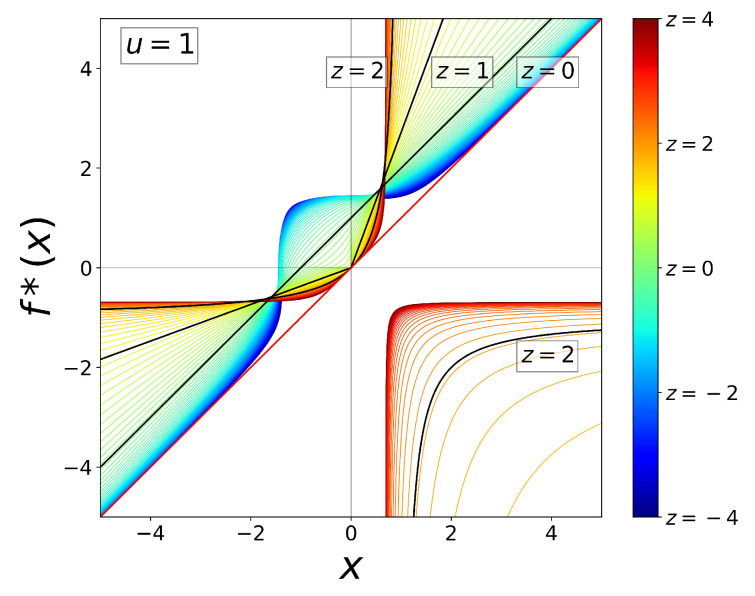
The RG fixed-point map $f^{*}\left(x\right)$ for the tangent bifurcation in Equation (3) shown for an extended range of values of the nonlinearity *z*. A two-branch map occurs for z>1 with one branch tangent with the identity line. When 0≤z≤2 , the left branch shows a cusp touching the identity line up to z≥1 . The branch at the right moves fast to infinity and dissapears at and below z≤1 . The map separates from the identity line for 0≤z≤1 and shows positive curvature around x=0 . When 0≤z , the shape of the map is inverted, showing now negative curvature around x=0 . See text.

**Figure 3:**
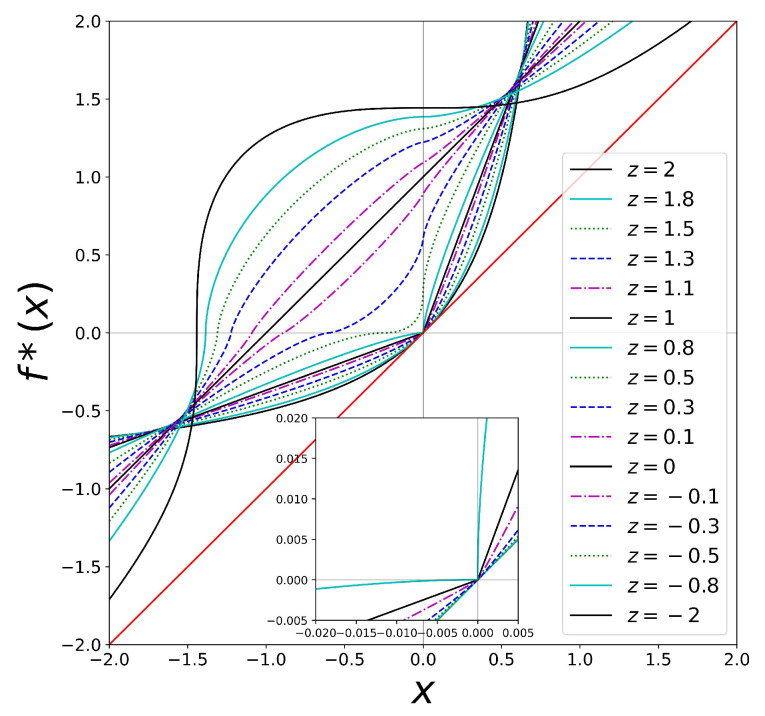
Similar to Figure 2, but showing more detail in the neighborhood of x=0 . The inset shows the cusp feature. See text.

Just as it is the case we have described above for rank distributions, here, we can also establish an exact analogy between the rate M(N) and the trajectories of the RG fixed-point map. Equations (12) and (13) are equivalent to Equations (14) and (15), respectively,

(14) $$\ln_{z}\left(x_{t}\right)=\ln_{z}\left(x_{0}\right)+ut$$

and

(15) $$x_{t}=x_{0}\exp_{z}\left({x_{0}}^{z-1}ut\right)$$

provided that we adopt the following identifications: N=t , $M_{0}=x_{0}$ , $M\left(N\right)=x_{t}$ , $\alpha^{-1}=z$ , ${\left(bN\right)}^{-1}=u$ , and $\mu \left(M\right)=\sum_{\tau =0}^{t}x_{\tau }$ . Except for a sign in the trajectory positions, these are the same that exhibit the equivalence between the stochastic process led by the parent distribution P(N) and the nonlinear iterated map f(x) [17,18,19]. That is, the trajectories of the RG fixed-point map for the tangent bifurcation reproduce the metabolism data of our formalism. However, there is an important issue here: the RG fixed-point map

(16) $$f^{*}\left(x\right)=x\exp_{z}\left(ux^{z-1}\right)$$

departs from the condition α=z=q≥2 and enters a previously unexplored regime. In Figure 2, we show $f^{*}\left(x\right)$ for a range of positive and negative values of α=z=q . In this figure, we observe in red/orange/yellow the known case z≥2 that consists of two branches, one that displays tangency with the identity function and the other at the bottom-right quadrant. Trajectories originated in this regime experience two different growth rates, slow growth at the left of the origin and superexponential growth at the right of the origin. If the RG fixed-point map is perturbed away from tangency, trajectories will exhibit intermittency, a nonlinear phenomenon we have employed in previous descriptions about complex systems from our nonlinear dynamical perspective. See Sections 2.3, 3.3, and 4.3 in Ref. [34]. When z≤2 tangency transforms into a cusp; the cusp is made of straight lines when the nonlinearity reaches z=1 , and consequently, the trajectories either decay exponentially ( x<0 ) or grow exponentially ( x>0 ). Below z=1 , the cusp separates from the identity line and becomes rounded as *z* distances from 1. The next limit case is z=0 , where the curvature of the map vanishes and trajectories grow linearly with time. As z<0 , a curvature develops opposite to the identity line, as is shown in Figure 2 in green-blue. Trajectories originated in this regime experience two different growth rates, fast growth near the origin and slower growth far from the origin, x≫0 . See also Figure 3.

## 4. Rich Gets Richer and Effortless Motion

We choose for the vegetable kingdom the universality class α=3 that we have identified to represent the rich-gets-richer principle or, in a network language, the preferential attachment processes [32]. In the preferential attachment network model [32], the connectivity (or degree) distribution is given by $P\left(L\right)=cL^{-3}$ , where *L* is the degree, or number of links stemming out of a node. This is equivalent to the parent distribution with α=3 we have chosen for the vegetable kingdom. This implies the metabolic rate

(17) $$R\left(M\right)=bM^{-1/3}$$

and the iterated map

(18) $$f\left(x\right)=x+ux^{-1/3}$$

from which we obtain (see Equation (15)) the RG fixed-point map $f^{*}\left(x\right)$ trajectories

(19) $$x_{t}=x_{0}\exp_{-1/3}\left(x_{0}^{-1/3-1}ut\right)\to {\left[4/3ut\right]}^{3/4},\;\;t\gg \left(3/4u\right)x_{0}^{4/3}.$$

**Figure 4:**
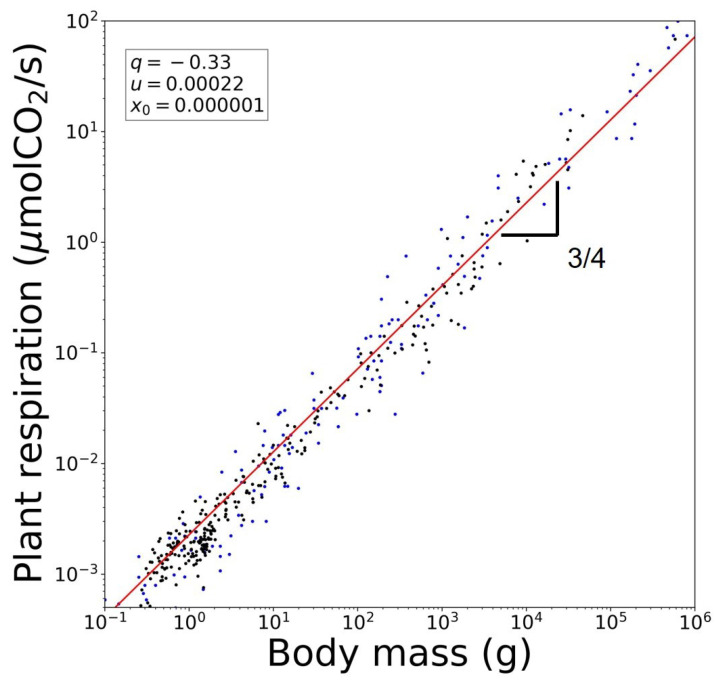
Reproduction of Kleiber’s law for the vegetable kingdom using data for plant respiration rates. Blue dots are data taken from [14]. Black dots are data taken from [35]. The red line is from Equation (19). See text.

That is, with t=N and $x_{t}=M\left(N\right)$ , we obtain, in accordance with Kleiber’s law, the scaling law $M\left(N\right)∼N^{3/4}$ for the metabolism M(N) . See Figure 4. Notice that we have considered for the vegetable kingdom the metabolic rate R(M) to be associated with ‘magnitude’ in our formalism in the sense previously described above.

Next, we consider the animal kingdom and choose the universality class α=4 that represents criticality, e.g., the absence of a (quadratic) curvature term in a Landau free energy [33]. In the Landau theory, the free energy functional is assumed to be an analytic function of the order parameter η . A typical example is a magnet, for which the free energy is a function only of even powers of η , and where the first (quadratic) term is a function of temperature *T*. At the phase transition, $T=T_{c}$ , a critical point, the quadratic term vanishes, making the next quartic term the dominant term. As a consequence, small displacements around the rest (or equilibrium) are costless.

However, as a difference with the above Equations (17)–(19), the metabolic rate R(M) is now considered to be associated with ‘frequency’, not ‘magnitude’, in the sense described in the previous sections. We can start our analysis of this case with a parent distribution $P\left(F\right)∼F^{-\beta }$ , β=4 and proceed to determine M(N) . However, as we know, this is equivalent to evaluating the functional inverse of the RG fixed-point map trajectories $x_{t+1}=f^{*}\left(x_{t}\right)$ that correspond to the map

(20) $$f\left(x\right)=x+ux^{1/4}.$$

We have (see Equation (14))

(21) $$t=u^{-1}\left[\ln_{1/4}x_{t}\;-\;\ln_{1/4}x_{0}\right]\to 4/3u^{-1}x_{t}^{3/4},\;\;x_{t}\gg x_{0}.$$

**Figure 5:**
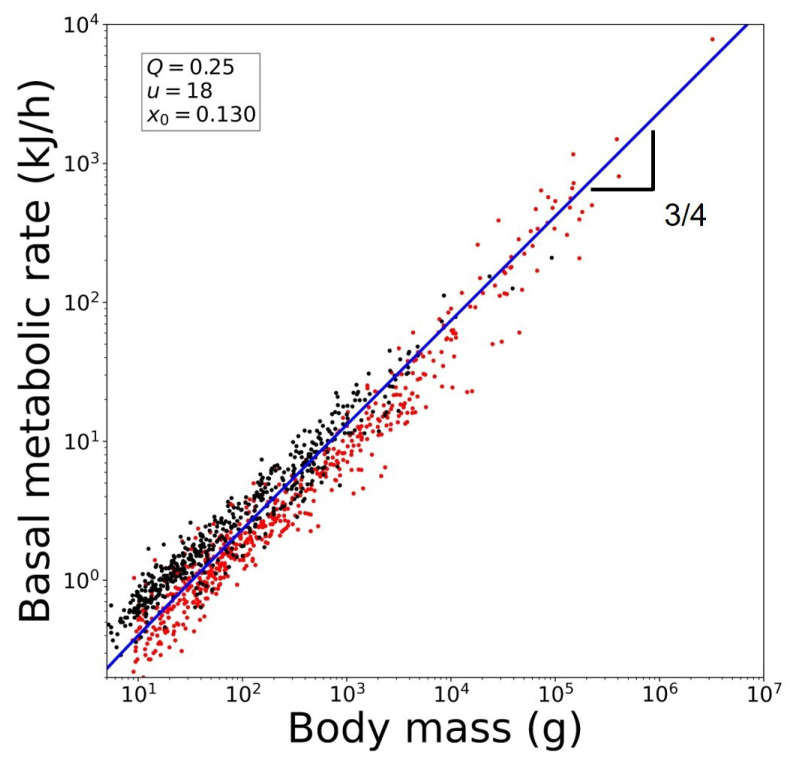
Reproduction of Kleiber’s law for the animal kingdom using data for basal metabolic rates. Mammals (red) [38] and avians (black) [39]. The blue line is from Equation (21). See text.

Now t=M(N) and $x_{t}=N$ , as the conjugate pair of the trajectory in Equation (19) with q=−1/3 , is the inverse function of the trajectory with Q=1/4 . That is, we obtain again, in accordance with Kleiber’s law, the scaling law $M\left(N\right)∼N^{3/4}$ for the metabolism M(N) of the animal kingdom. See Figure 5.

The occurrence of the same power-law exponent 3/4 for the metabolism of the two kingdoms involved in Kleiber’s law, plants and animals, appears as one instance in the locus of conjugate values for the pairs of deformation exponents (q,Q) for the *q*-exponential and the *Q*-logarithmic functions shown in Figure 1.

Another possible example of a conjugate pair (q,Q) that involves a tight relationship between ‘magnitudes’ and ‘frequencies’ is that of river flow. In this case, we have Hack’s law that relates river lengths with flow through transverse sections [36]. Hack’s law shows the scaling of the largest upstream length $L_{\max}$ with its total cumulative area $A_{\max}$ , $L_{\max}∼A_{\max}^{h}$ , where h∼0.57 [37]. This river structure can be theoretically approximated, among other possibilities [37], by the ‘directed network model’ [37] that complies with (q=−1/2,Q=1/3) and yields ζ=h=2/3 [37].

## 5. Summary and Discussion

We have extended our nonlinear dynamical approach that reproduces real data for rank distributions [17,18,19], functions that decay either exponentially or as a power law (e.g., Zipf’s law), to other measurable quantities, like dissipation rates as functions of mass or volume of organisms (geological, biological, urban), functions that increase exponentially or as power laws (e.g., Kleiber’s law). We have emphasized the presence of universality classes (given by the exponent values α of the parent or source distributions $P\left(N\right)∼N^{-\alpha }$ ). These exponent values coincide with the nonlinearity exponent *z* of the iterated map f(x) equivalent to P(N) and with values of the deformation parameter *q* of the deformed exponential in the RG fixed-point map $f^{*}\left(x\right)$ ruling in the background, α=z=q . We have, in particular, focused on the occurrence of conjugate pairs of deformation values (q,Q) that display the same power-law exponent ζ (within appropriate intervals of the independent variable: rank *k*, iteration time *t*, mass or volume *N*) for the *q*-exponential function and its functional inverse, the *Q*-logarithm. See Figure 1. One important instance is that of Zipf’s law (q=2,Q=2) , a situation in which our approach is capable of distinguishing between magnitude-rank and frequency-rank distributions [17]. Additionally, significantly, at these deformation values (q=2,Q=2) , the RG fixed-point map $f^{*}\left(x\right)$ is at a borderline (signaled, e.g., by the divergence of prime number reciprocals [19]), where the shape of $f^{*}\left(x\right)$ undergoes an important transformation (see Figure 2 and Figure 3).

The transformation undergone by $f^{*}\left(x\right)$ at z=2 is precisely the feature that we have taken advantage of to extend our approach from rank distributions to the description of scaling laws for quantities such as rates of dissipation as a function of system size (e.g., metabolic rates). Figure 2 shows $f^{*}\left(x\right)$ for a range of values of its nonlinearity *z*. When z>1 , the RG map has two branches, one of them tangent with the identity line. The map develops a cusp at x=0 as z→1 , while for z≤1 , the second branch vanishes. The cusp becomes disconnected with the identity line just below z=1 and from there shows an indentation (positive curvature) around x=0 . The shape of $f^{*}\left(x\right)$ transforms again at z=0 when the curvature near x=0 changes sign.

What we have done here is to show that the trajectories produced by the RG fixed-point map, in one case 0<z<1 and in the other case −1<z<0 , are capable of quantitatively reproducing the metabolism data involved in Kleiber’s law. Our reasoning started by choosing two universality class exponent values: α=z=q=3 (for ‘magnitudes’, representing ‘rich gets richer’) and α=z=q=4 (for ‘frequencies’, representing null cost for small displacement motion). With these values, we formulated the RG fixed-point map and its trajectories that yield us the desired function M(N) , with metabolism being a function of individual mass or volume *N*. The chosen values α=3 and α=4 became, in our formalism, one pair of conjugate values (q=−1/3,Q=1/4) that have the property of producing the same value of the scaling exponent 3/4 in $M\left(N\right)∼N^{3/4}$ , or Kleiber’s law. See Figure 1, Figure 4 and Figure 5.

Recently [25], we have demonstrated that the trajectories of all RG fixed-point maps for the three known routes to chaos (intermittency, period doubling, and quasi-periodicity [24]) can be couched in the statistical–mechanical language of the (discrete time) Landau–Ginzburg (LG) equation. Additionally, the associated Lyapunov function [40] is precisely the expression for the Tsallis entropy [25]. Equation (10) is a particular case of the LG equation used to describe the most probable evolution of processes in statistical–mechanical systems. See [25] and references therein. The role of time *t* in the LG equation in Equation (10) is taken by the mass *N*, while *M* is a macroscopic variable relevant to the process described. For the plant kingdom, the differential equation’s driving force is the power law $M^{-1/3}$ . This driving force is the (functional) derivative of the Lyapunov function. This function represents a generalized thermodynamic potential and evolves monotonically as *t*, or *N*, increases along the solution of the LG equation [25]. In the case of Equations (10) and (17), it is given by

(22) $$S_{q}=\ln_{q}M,\;\;q=1/3.$$

The Tsallis entropy above corresponds to a uniformly distributed set of events. It merely states that every time unit that makes up the characteristic time $T=R^{-1}$ for an organism of mass *N* equally contributes to its total value T(N) . Furthermore, for large *N*

(23) $$S_{q}∼M{\left(N\right)}^{4/3}={\left[N^{3/4}\right]}^{4/3}=N;$$

i.e., the Tsallis entropy in Equation (22) is extensive for the mass *N*. A parallel argument for the animal kingdom, which takes into account that the conjugate pair (q,Q) involves functions inverse to each other, leads too to an extensive Tsallis entropy. Moreover, considering that data for Kleiber’s law consist of a list (or lists) of measured values of metabolic rates for a set (or sets) of species, we can write the rate equation in Equation (10) for discrete time. Clearly, this is the nonlinear iterated map in Equation (18) that, under the requirement that functional composition is equivalent to rescaling, leads to the RG fixed-point map $f^{*}\left(x\right)$ in Equations (3) or (16) with z=−1/3 , and similarly with Equation (20).

It is important to emphasize that our approach leads to analytical closed-form expressions for the metabolic rate R(M) in terms of the *q*-exponential and *q*-logarithmic functions in Equations (19) and (21). The power laws with the exponent 3/4 correspond to the asymptotic, large *N*, behavior of these expressions. The full set of properties of R(M) includes consideration of the entire positive real number interval, small and large *N*. The small *N* conduct of the *q*-deformed functions may explain the observed 2/3 exponent in some data samples. Meanwhile, finite-size effects present for large *N* can be quantitatively reproduced via the shift of the maps involved away or towards the identity function, as it has been done for the rank distributions [17,18,19]. Therefore, the study presented here is yet another example of a complex system problem where the Tsallis generalized statistical mechanics provide pertinent results. Other issues addressed that involve Tsallis generalized entropy and related quantities are [34] within condensed matter physics: The formation of glasses, the transformation of a conductor into an insulator, and critical point fluctuations; concerning complex systems problems, the phenomenon of self-organization and the development of diversity (biological or social, like languages); and, as described here, the comprehension of empirical laws, like those relating to the universality of ranked data or the power-law scalings present in allometry. A common feature in all these cases is that access to their configurational space is severely hindered to a point where the allowed configurational space has a vanishing measure with respect to the initial setup [25]. This restriction is naturally provided by the attractors at the transitions to chaos present in the nonlinear dissipative maps employed to model these subjects [25].

As a finishing remark, we would like to bring attention to a set of curious circumstances where low-dimensional nonlinear dynamics have inadvertently been used to model complex systems. Such is the case of the “cobweb theorem” in economics [41,42], where successive iterations are employed to model actual price dependence on past offer. The next instance is in the study of biological rhythms [43], where cobweb plots are referred to as “zig-zag lines from cause to effect”. These encounters with nonlinear dynamics occurred years before the subject was more formally advanced with the use of the RG technique as in the works of Feigenbaum [44] and Hu and Rudnick [22], but point towards its use in the modeling of complex phenomena. Ours is a quantitative attempt to establish a methodology based on nonlinear dynamics to study complex systems

We wish a joyful 80th birthday to Constantino Tsallis.

## Data Availability

Data are available upon request.
